# eIF2α signaling regulates autophagy of osteoblasts and the development of osteoclasts in OVX mice

**DOI:** 10.1038/s41419-019-2159-z

**Published:** 2019-12-04

**Authors:** Jie Li, Xinle Li, Daquan Liu, Kazunori Hamamura, Qiaoqiao Wan, Sungsoo Na, Hiroki Yokota, Ping Zhang

**Affiliations:** 1 Department of Anatomy and Histology, School of Basic Medical Sciences, Tianjin Medical University, 300070 Tianjin, China; 2 Key Laboratory of Hormones and Development (Ministry of Health), Tianjin Key Laboratory of Metabolic Diseases, Tianjin Medical University, 300070 Tianjin, China; 3 Department of Biomedical Engineering, Indiana University Purdue University Indianapolis, Indianapolis, IN 46202 USA; 4 Tianjin Key Laboratory of Spine and Spinal Cord, Tianjin Medical University, 300052 Tianjin, China

**Keywords:** Stem-cell differentiation, Osteoporosis, Experimental models of disease

## Abstract

Bone loss in postmenopausal osteoporosis is induced chiefly by an imbalance of bone-forming osteoblasts and bone-resorbing osteoclasts. Salubrinal is a synthetic compound that inhibits de-phosphorylation of eukaryotic translation initiation factor 2 alpha (eIF2α). Phosphorylation of eIF2α alleviates endoplasmic reticulum (ER) stress, which may activate autophagy. We hypothesized that eIF2α signaling regulates bone homeostasis by promoting autophagy in osteoblasts and inhibiting osteoclast development. To test the hypothesis, we employed salubrinal to elevate the phosphorylation of eIF2α in an ovariectomized (OVX) mouse model and cell cultures. In the OVX model, salubrinal prevented abnormal expansion of rough ER and decreased the number of acidic vesiculars. It regulated ER stress-associated signaling molecules such as Bip, p-eIF2α, ATF4 and CHOP, and promoted autophagy of osteoblasts via regulation of eIF2α, Atg7, LC3, and p62. Salubrinal markedly alleviated OVX-induced symptoms such as reduction of bone mineral density and bone volume fraction. In primary bone-marrow-derived cells, salubrinal increased the differentiation of osteoblasts, and decreased the formation of osteoclasts by inhibiting nuclear factor of activated T-cells cytoplasmic 1 (NFATc1). Live cell imaging and RNA interference demonstrated that suppression of osteoclastogenesis is in part mediated by Rac1 GTPase. Collectively, this study demonstrates that ER stress-autophagy axis plays an important role in OVX mice. Bone-forming osteoblasts are restored by maintaining phosphorylation of eIF2α, and bone-resorbing osteoclasts are regulated by inhibiting NFATc1 and Rac1 GTPase.

## Introduction

Osteoporosis is one of the common skeletal diseases, which presents a systemic impairment of bone mass and micro-architecture. Its medical and socioeconomic impacts, particularly those with postmenopausal osteoporosis in the aging population, are expected to sharply increase^1^. Postmenopausal osteoporosis can be treated by a variety of drugs, including anti-resorptive agents, anabolic agents, and emerging monoclonal therapies targeted to sclerostin^2–5^. None of them, however, provide an ideal therapeutic option because of their side effects and/or limited efficacy.

It is recently reported that the stress to the endoplasmic reticulum (ER) is closely related to the progression of skeletal disorders, including osteoporosis^6^. The ER stress is a general term with varying stress sources that impede the regular ER function^7^. Its evolutionarily conserved pathway leads to the unfolded protein response (UPR)^8^, in which protein kinase-like endoplasmic reticulum kinase (PERK) acts as a sensor for accumulation of unfolded proteins in the ER lumen of mammalian cells. It phosphorylates the subunit of the translation initiation factor, eukaryotic translation initiation factor 2 alpha (eIF2α), which suppresses general protein production except for the selective stress responsive factors such as activating transcription factor 4 (ATF4)^9^. Our previous study indicated that the ER stress plays an important role in the development of disuse osteoporosis^10^. In postmenopausal osteoporosis, the common type of osteoporosis, the mechanism by which the ER stress regulates bone homeostasis has not been elucidated.

Autophagy is a process of disassembling cellular components to cope with various cellular malfunctions, including UPR^11,12^. It is an intracellular degradation mechanism in eukaryotic cells that transports damaged cytoplasmic components to a lysosome for degradation and recycling^13^. Autophagy is reported to be involved in the regenerative function of mesenchymal stem cells (MSCs) in bone marrow, as well as the progression of osteoporosis^14^. Previous studies have shown that deficiency in autophagy in osteoblasts decreases their mineralizing capacity and leads to a low bone mass phenotype. Particularly, the autophagy proteins, Atg7, is needed for mineralization of an osteoblastic cell line^15^. Its deficiency impedes osteoblast mineralization, while its reconstitution is shown to restore skeletal balance^16^. However, the role of autophagy and Atg7 in postmenopausal osteoporosis remains unclear.

Salubrinal is a 480-Da synthetic agent (C_21_H_17_Cl_3_N_4_OS), which is known to inhibit the de-phosphorylation of eIF2α^17^. Its effects on the differentiation of bone-marrow-derived cells to osteoclasts and osteoblasts, is not well understood. Furthermore, the mechanism eIF2α interacts with Rho family GTPases such as Rac1, which play important roles in bone formation and resorption^18,19^, remains elusive.

An ovariectomized (OVX) mouse model mimics the increased bone turnover induced by menopause in humans^20,21^. Herein, we investigated the effects of administration of salubrinal to the OVX mice, with a focus on salubrinal’s dual role in regulating osteoblasts and osteoclasts. We also evaluated the effects of salubrinal in vitro using primary bone-marrow-derived cells, as well as osteoblastic and pre-osteoclastic cell lines.

## Materials and methods

### Animals and agents

Animal use was approved by the Ethics Committee at Tianjin Medical University. We used 123 C57BL/6 female mice in total (~16 wks, Animal Center of Academy of Military Medical Sciences, China). Under pathogen-free conditions, 4–5 mice were housed per cage, and they were maintained at 25 °C on a 12-h light/dark cycle, with standard rodent chow and water ad libitum.

We purchased a murine RANKL (receptor activator of nuclear factor kappa-B ligand) and murine M-CSF (macrophage-colony stimulating factor) from PeproTech, and Dulbecco’s Modified Eagle’s Medium (DMEM), Minimum Essential Medium Alpha (MEM-α), fetal bovine serum (FBS), penicillin, streptomycin, and trypsin from Invitrogen (Carlsbad, CA, USA). Other agents were obtained from Sigma (St. Louis, MO, USA) unless otherwise stated.

### Cell culture

MC3T3-E1 cells were obtained from Chinese Academy of Sciences Cell Bank. MC3T3-E1 cells differentiation were initiated in α-MEM containing 10% FBS, 100 U/mL penicillin, 100 μg/mL streptomycin, 10 mM β-glycerophosphate, and 50 μg/mL ascorbic acid^22,23^. ALP and Alizarin Red S staining was conducted to confirm osteoblasts before following experiment.

### OVX mouse model

The mice were randomly divided into three groups: sham control group (Sham, *n* = 41), OVX group (OVX, *n* = 41), and salubrinal-treated OVX group (OVX + Sal, *n* = 41). Anesthesia was induced using 1.5% isoflurane (IsoFlo, Abbott Laboratories) with a rate of 0.5–1.0 L/min. After shaving the hair in the dorsal mid-lumbar area, the skin was cleaned and a 20-mm midline dorsal skin incision was made. After excision of a pair of ovaries, the wound was closed by suturing^24,25^. The sham control mice received the same procedure but no removal of the ovaries. In 4 weeks, the OVX + Sal group was given daily subcutaneous injection of salubrinal (Tocris Bioscience). The vehicle was 49.5% PEG400 with 0.5% Tween 80, and salubrinal’s dose was 1 mg/kg body weight. The OVX group was given an equal volume of vehicle^10^. Mice were sacrificed in week 8.

### Bone mineral density (BMD) and bone mineral content (BMC)

BMD (g/cm^2^) and BMC (g) were determined for a whole body on live mice, lumbar spines, femurs, and tibia using peripheral dual-energy X-ray absorptiometry (pDEXA)^26^, and ROI analysis was conducted.

### Micro-computed tomography

A Scanco vivaCT 40 (Scanco Medical AG, Bassersdorf, Switzerland) was employed for micro-computed tomography (µCT) as previously reported^27^. Using 6-μm pixel size, we set an X-ray source at 60 kV and scanned the excised left distal metaphysis of the femurs. We focused on a ~0.5 mm proximal region in the most distal part of the growth plate. Base on three-dimensional (3D) images, we determined parameters such as BV/TV (bone volume fraction, %), Tb.N (trabecular number, 1/mm), Tb.Th (trabecular thickness, μm), and Tb.Sp (trabecular bone spacing, μm).

### Histology and histomorphometry

The femur was fixed in 10% neutral buffered formalin for 2 days, followed by decalcification in 14% EDTA for 2 weeks. The paraffin-embedded samples were cut into 5-μm-thick coronal sections and stained with hematoxylin-eosin (H&E). On the proximal side of the growth plate, we determined B.Ar/T.Ar (bone area/total area) within 2.6-mm^2^ sample area.

The differentiation and maturation of osteoclasts were evaluated with tartrate-resistant acid phosphatase (TRAP) staining^28^. We also employed MacNeal’s staining for identification of osteoblasts^29^. The number of osteoblasts was normalized using the trabecular bone surface, and histomorphometry of the distal metaphysis of the femur were performed. All samples were blinded for quantitative analysis.

For evaluation of bone formation, we conducted calcein labeling with intraperitoneal injection of 10 mg/kg calcein on day 7 and 2 days prior to sacrifice^30,31^. The femur samples were prepared in LR white acrylic resin, and 15-μm sections were cut for determination of dynamic parameters, such as mineral apposition rate (MAR; interlabel width/day), mineralizing surface/bone surface [MS/BS: (double labeled surface + single-labeled surface/2)/BS], and bone formation rate [BFR/BS: MAR × MS/BS/100]^32^.

### Electron microscopic analysis

The femurs and MC3T3-E1 osteoblast-like cells were imaged using electron microscopy^33–35^. After sample fixation, decalcification was conducted in 14% EDTA for 2 weeks. Post-fixation was conducted in 1% osmium tetroxide for 1 h. After dehydration, they were infiltrated in epoxypropane, followed by embedding in EPON812. We generated ~50 nm sections and imaged with a model 7500 electron microscope (Hitachi). Fifty sections were made for each sample and the number of autophagic vacuoles was identified. A MEGAVIEW camera was used to capture the images of osteoblasts. The rough ER area percentages in the osteoblastic cytoplasm and the number of intracellular autophagosomes in every 10 fields were determined using Cellsense standard software.

### Immunohistochemistry assay

For immunohistochemical analysis, femoral sections were incubated with primary antibodies against ATF4 and CHOP (Cell Signaling, Danvers, MA, USA) at 4 °C overnight. An immunohistochemical kit and 3, 3′-diaminobenzidine (DAB) (ZSGB-BIO, Beijing, China) substrate kit were used according to the manufacturer’s protocol. Approximately 6–8 sections were obtained from each sample, and the same region was analyzed from all samples^36,37^.

### ELISA assay

Blood samples were collected from mice. A bone formation markers, amino terminal propeptide of type I collagen (PINP), and a bone resorption marker, tartrate-resistant acid phosphatase type 5b (TRACP-5b), were detected according to the instructions of manufacturer^16,38^.

### Immunofluorescence analysis of LC3

Immunofluorescence analysis was conducted as described previously^39^. MC3T3-E1 cells were cultured with DMSO (vehicle), 5 μM salubrinal, 100 ng/ml tunicamycin, 3-MA, or 200 nM rapamycin for the positive control for 48 h. They were incubated with LC3 antibody for labelling autophagosomes, followed by incubation with fluorescent antibody in dark. DAPI was employed to counterstain the nuclei. The experiment was conducted in triplicate.

### CFU-F (colony-forming unit-fibroblasts), CFU-OBL (colony-forming unit-osteoblasts), and osteoblast mineralization

We conducted the CFU-F assay and evaluated the colony formation ability of fibroblast-like MSCs^40,41^. We counted CFU-F colonies with more than 50 cells, and we excluded the clusters that did not present fibroblast-like morphology.

In the CFU-OBL assay, we isolated bone-marrow-derived cells and plated them at 2 × 10^6^ cells/ml in osteogenic differentiation medium (MesenCult proliferation kit)^29^. The medium was exchanged every other day for 2 weeks, followed by alkaline phosphatase (ALP) staining including counterstaining with Mayer's Hematoxylin.

A mineralization assay with primary osteoblasts was conducted^42^. After fixation in 100% ethanol on ice for 1 h, cells were stained with 1% Alizarin red S dye (pH 4.1) and rinsed five times with deionized water.

### Osteoclast differentiation from bone-marrow-derived cells

We harvested bone-marrow-derived cells from the iliac, femur, and tibia in Iscove’s MEM (Gibco-Invitrogen)^43^. Cells were isolated using low-density gradient centrifugation, and grown in α-MEM, containing 10% FBS, 20 ng/ml RANKL, and 30 ng/ml M-CSF. The medium was exchanged on day 4 with α-MEM consisting of 10% FBS, 60 ng/ml RANKL, and 30 ng/ml M-CSF, and cells were cultured for 3 additional days^44^. We evaluated osteoclast differentiation using bone-marrow-derived cells in the presence and absence of salubrinal^45^, and evaluated adherent cells using a tartrate-resistant acid phosphate (TRAP)-staining kit on day 6. We identified TRAP-positive osteoclasts with more than three nuclei, and determined the surface area with mature osteoclasts.

### Osteoclast migration and adhesion

Using a transwell assay, we evaluated migration of osteoclasts as described previously^43^. We also evaluated osteoclast adhesion using precursor cells using a vitronectin-coated plate^44^. After 30 min incubation, cells were rinsed and fixed with 4% paraformaldehyde, and the number of cells adherent to α_v_β_3_ was counted after crystal violet staining.

### Quantitative PCR and western blot analysis

Quantitative PCR was conducted for RAW264.7 pre-osteoclast cells and MC3T3-E1 osteoblast-like cells (C14 clone), which were grown in MEM with 10% FBS and antibiotics^46^. Osteoclastogenesis of RAW264.7 cells were induced by incubation with 20 ng/ml of RANKL. The PCR primers were: NFATc1 (5′-GGT GCT GTC TGG CCA TAA CT-3′ and 5′-GCG GAA AGG TGG TAT CTC AA-3′); TRAP (5′-GCA AAT ACC CAC AGT TCC GC-3′ and 5′-AAG ACT CCC AGC GTC ACG TC-3′); cathepsin K (5′-CAG CTT CCC CAA GAT GTG AT-3′); and GAPDH (5′-TGC ACC ACC AAC TGC TTA G-3′ and 5′-GGA TGC AGG GAT GAT GTT C-3′) as an internal control.

For western blot analysis, we isolated proteins from femurs, bone-marrow-derived cells, RAW264.7 cells, and MC3T3-E1 cells. Bilateral femurs were obtained and triturated in liquid nitrogen. Briefly, the harvested samples were placed in a pre-cooled mortar, and liquid nitrogen was added for condensing the sample. After liquid nitrogen volatilization, the nucleus pulposus was ground into a powder using a pestle. During grinding, liquid nitrogen was added until the sample had been sufficiently ground. Antibodies specific to Bip (Affinity BioReagents), cathepsin K, NFATc1, and TRAP (Santa Cruz), ALP (Proteintech), eIF2α, p-eIF2α (Cell Signaling), Rac1 (Millipore), Atg7 (Abcam), LC3, p62 (Medical & Biological Laboratories), and β-actin (Sigma) were employed.

### Fluorescence-based osteoclast activity assay

We evaluated osteoclast activity using a bone resorption assay kit (Cosmo Bio). In brief, RAW264.7 cells were cultured for 7 days on a plate coated with fluoresceinamine-labeled calcium phosphate in the presence and absence of RANKL and salubrinal. Fluorescence intensity of the culture medium was measured using excitation/emission wavelengths at 485/535 nm.

### Knockdown of eIF2α, Atg7, and Rac1 by siRNA

RNA interference was conducted to osteoblasts differentiated from bone-marrow-derived cells, MC3T3-E1 cells and RAW264.7 cells using siRNAs specific to eIF2α, Atg7, and Rac1 (Life Technologies). The selected target sequences were: Atg7, 5′-AUU AGA GGG AUG CUC UCA GTT-3′; eIF2α, 5′-AAG CUA CUU CAA UAU CUG CTT-3′; Rac1, 5′-GCA UUU CCU GGA GAG UAC A -3′; and non-specific control (Scrambled siRNA 5′-ACG UGA CAC GUU CGG AGA TTA-3′, Life Technologies). Cells were transiently transfected using Lipofectamine RNAiMAX in Opti-MEM I medium (Life Technologies). The transfection medium was replaced in 6 h, and the silencing efficiency was evaluated using Western blot in 48 h after transfection according to the manufacturer’s instruction.

### Fluorescence resonance energy transfer (FRET) imaging

To evaluate the effect of salubrinal on Rac1 activity, live cell imaging was conducted using a Rac1 FRET biosensor in RAW264.7 cells. Activation of Rac1 results in conformational changes of the biosensor that shift emission signal from cyan fluorescent protein (CFP) to yellow fluorescent protein (YFP). Rac1 activity is thus evaluated by an intensity ratio of YFP/CFP in individual cells^47,48^. We acquired fluorescent time-lapse images at an interval of 5 min, and FRET images (YFP/CFP emission ratio) were depicted by NIS-Elements software (Nikon).

### Statistical analysis

Data were shown as mean ± SEM, and statistical significance was tested using one-way ANOVA. We conducted a post-hoc test using Fisher’s protected least significant difference. In FRET data analysis, we determined the mean activation level of Rac1 at each time point and conducted one-way ANOVA with Dunnett’s post-hoc test. Statistical significance of the salubrinal-injected sample to the control sample was examined using a paired *t*-test. All comparisons were two-tailed and statistical significance was assumed at *P* *<* 0.05. The single, double, and triple asterisks represent *P* *<* 0.05, 0.01, and 0.001, respectively.

## Results

### Attenuation of OVX-driven effects in salubrinal-administered mice

**Fig. 1:**
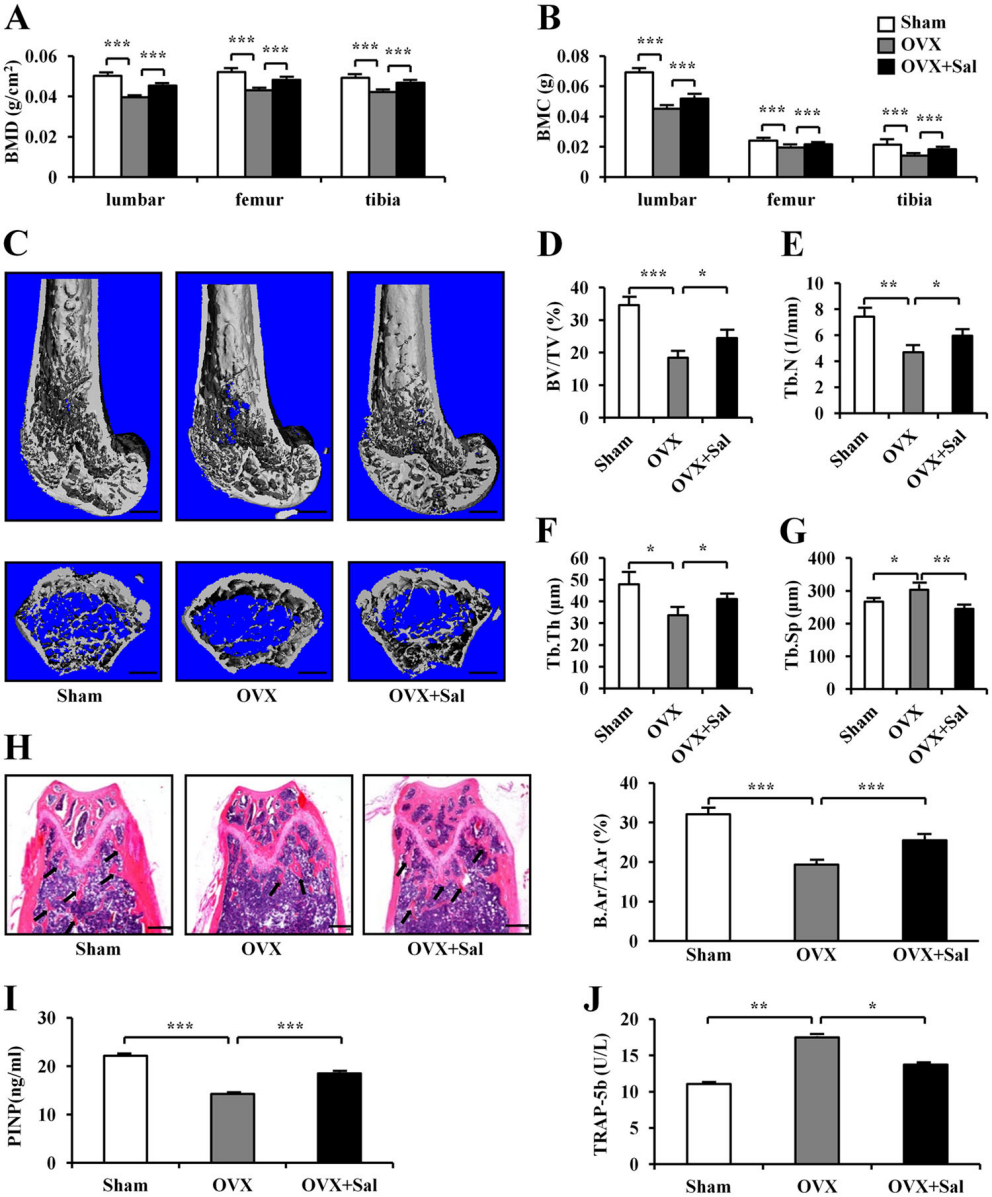
Effects of ovariectomy and salubrinal on BMD, BMC, micro-architecture, serum PINP, and TRACP 5b. **a, b** Suppression of OVX-induced reduction of BMD and BMC, in the lumbar spine, femur, and tibia. Salubrinal increased BMD and BMC, compared to OVX in the lumbar spine, femur and tibia. **c**–**f** Representative µCT reconstructed femurs in the longitudinal (top) and transverse (bottom) cross-sections after 4-week treatment with salubrinal (**c**), salubrinal-driven suppression of OVX-induced loss of femoral BV/TV (**d**), femoral trabecular number (Tb.N) (**e**), and femoral trabecular thickness (Tb.Th) (**f**), Salubrinal-driven suppression of OVX-induced increase in femoral trabecular spacing (Tb.Sp) (**g**) (Bar = 500 μm). **h** B.Ar/T.Ar ratio in the distal metaphysis of the femurs (Bar = 500 μm). Trabecular bones were indicated by the arrows. **i, j** Serum PINP and TRACP 5b analyses (*n* = 5). The asterisks (*, **, and ***) represent statistical significance at *P* *<* 0.05, *P* *<* 0.01, and *P* *<* 0.001, respectively (*n* = 12).

Compared to the sham group, OVX mice significantly decreased BMD and BMC not only in the lumbar spine, but also in the femur and tibia. Compared to OVX mice, salubrinal-treated OVX mice exhibited a statistically significant increase in BMD and BMC not only in the lumbar spine, but also in the femur and tibia (all *P* *<* 0.001; Fig. 1a, b).

Compared to the sham group, micro-CT imaging of the distal metaphysis of the femurs (Fig. 1c) indicated that the values of BV/TV (*P* *<* 0.001), Tb.N (*P* *<* 0.01), and Tb.Th (*P* *<* 0.05) in OVX mice were decreased, with an increase in Tb.Sp (*P* *<* 0.05). In addition, BV/TV was altered from 18.41 ± 1.87% (OVX) to 24.46 ± 2.57% (OVX + Sal) (*P* *<* 0.05; Fig. 1d). The trabecular number was elevated by 27.35% from 4.68 ± 0.49 (OVX) to 5.96 ± 0.42 (OVX + Sal) (*P* *<* 0.05; Fig. 1e), and the femoral trabecular thickness was increased by 22.19% with salubrinal (*P* *<* 0.05; Fig. 1f). However, the femoral trabecular spacing was decreased by 23% with salubrinal (*P* *<* 0.001; Fig. 1g).

To evaluate salubrinal’s effect on bone architecture, the bone area/total area (B.Ar/T.Ar) in the distal metaphysis of the femurs was determined using H&E staining (Fig. 1h). OVX mice presented a reduction in B.Ar/T.Ar compared with the sham control mice (*P* *<* 0.001; 32.05 ± 1.50% in the sham control mice, and 19.31 ± 0.82% in the OVX mice). However, administration of salubrinal significantly restored B.Ar/T.Ar (*P* *<* 0.001; 25.50 ± 1.03% in salubrinal-treated OVX mice).

To evaluate bone resorption and bone formation, serum biomarkers were used. Compared to the sham group, we observed a reduction in PINP (*P* *<* 0.001; Fig. 1i), a circulating marker of bone formation, and an increase in TRACP-5b, a bone resorption marker, in serum from OVX mice (*P* *<* 0.01; Fig. 1j). Administration of salubrinal significantly restored OVX-induced decrease in PINP (*P* *<* 0.001; Fig. 1i) and the increase in the serum level of TRACP-5b (*P* *<* 0.05; Fig. 1j).

### Salubrinal-driven differentiation of osteoblast in vivo and in vitro

**Fig. 2:**
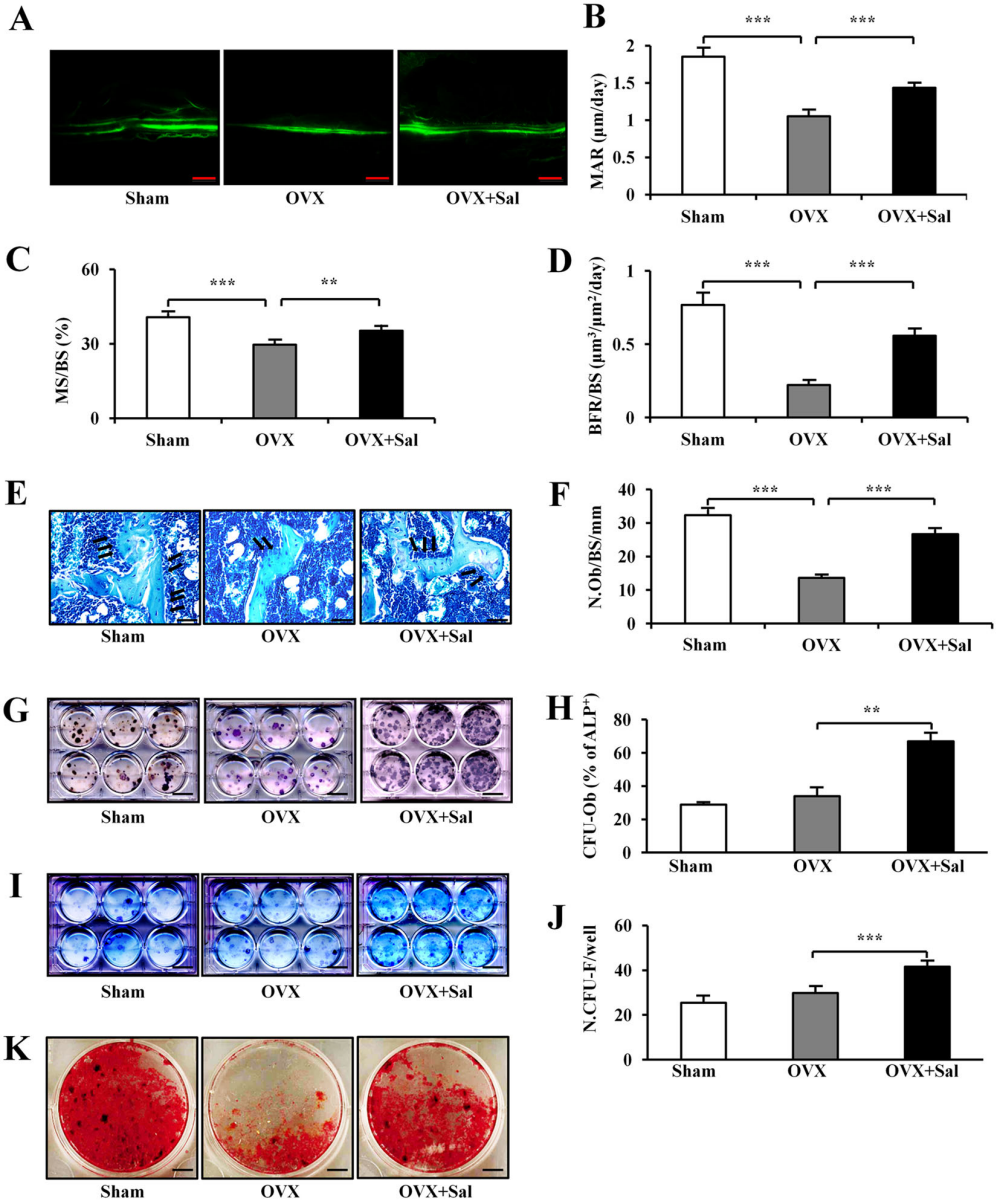
Effects of salubrinal on osteoblast differentiation. **a**–**d** Calcein-labeled cortical femoral bones. Mineral apposition rate (MAR), MS/BS and BFR/BS was calculated (Bar = 100 μm). **e, f** Salubrinal-induced increase in the osteoblast numbers in OVX mice. The microphotographs represent the three groups of MacNeal’s staining (Bar = 50 µm). Osteoblasts, located on the trabecular bone surface, were indicated by the arrows. **g, h** Comparison of CFU-F. **i, j** Comparison of CFU-OBL. Bone-marrow-derived cells were isolated from three groups of mice (Sham, OVX and salubrinal-injected OVX mice) (Bar = 2 cm). **k** Osteoblast mineralization (Alizarin Red staining) were determined in bone-marrow stem cells after they were cultured in the osteoblast inducing medium (Bar = 2 mm). The representative photographs are shown. The asterisks (** and ***) represent statistical significance at *P* *<* 0.01 and *P* *<* 0.001, respectively (*n* = 20).

In the present mouse model, we examined the parameters for bone formation in the long bones using calcein labeling. Compared to the sham group, OVX reduced BFR in cancellous bone by the combination of reduction in MS/BS and MAR (all *P* *<* 0.001). However, salubrinal restored BFR (*P* *<* 0.001) in cancellous bone by combined promotion of MS/BS (*P* *<* 0.01) and MAR (*P* < 0.001; Fig. 2a–d).

MacNeal’s staining revealed that compared to the sham group, OVX mice reduced the number of osteoblasts on a trabecular bone surface (*P* *<* 0.001). However, administration of salubrinal markedly elevated the number of osteoblasts (*P* *<* 0.001; Fig. 2e, f). Cells derived from the salubrinal-treated OVX mice elevated the number of colonies in CFU-F (*P* *<* 0.01 for OVX vs OVX + Sal) and CFU-OBL (*P* *<* 0.001 for OVX vs OVX + Sal), compared to cell cultures derived from the sham-operated and OVX mice (Fig. 2g–j). The result with alizarin red staining showed that the OVX group decreased the number of mineralized nodules compared to the sham control group, while salubrinal-treated mice promoted mineralization of osteoblasts (Fig. 2k).

### Salubrinal-driven suppression of ER stress and activation of autophagy in osteoblasts

**Fig. 3:**
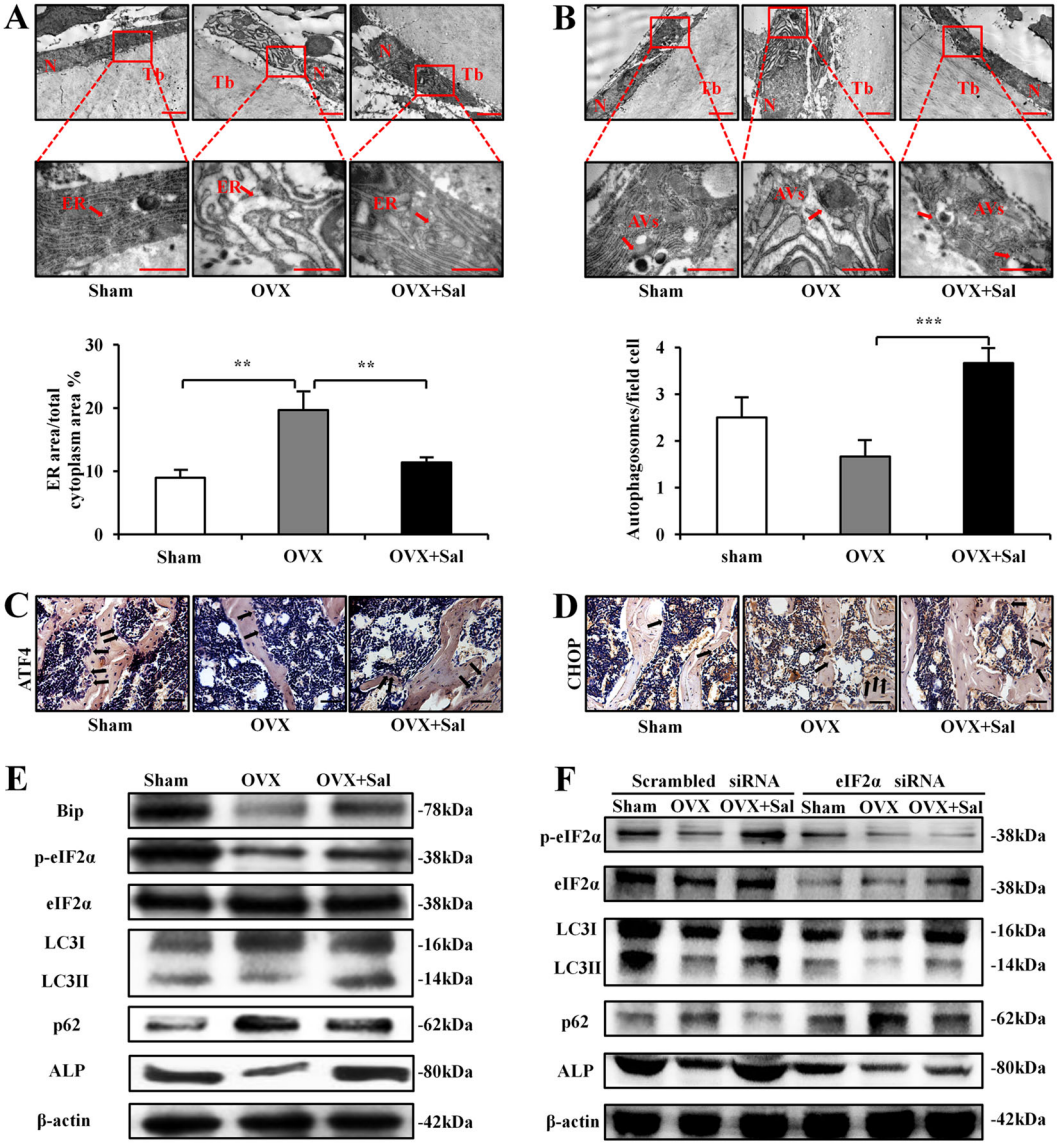
Regulation of ER stress and autophagy by salubrinal. **a, b** Electron micrographs, indicating that ultrastructural changes of the rough endoplasmic reticulum (ER) and autophagy in osteoblasts from OVX mice after treatment with salubrinal. Quantitative analyses of the rough ER areas in the cytoplasm of osteoblasts. The percentages of the rough ER area were measured. The rough ER were indicated by the red arrows (**a**). Representative electron micrographs of accumulation of autophagic vacuoles in osteoblast cells. Quantitation of autophagosomes in osteoblasts. The autophagosomes were indicated by the red arrows (**b**). Of note, N indicated the nucleus. Tb indicated the trabecular bone, and AVs indicated the acidic vesicular (Bar = 2 μm on the upper, and Bar = 1 μm on the bottom). **c, d** Immunohistochemistry staining of ATF4 and CHOP of osteoblasts in femurs were conducted (Bar = 50 μm). **e** Representative images of Western blot in three different groups in vivo. The levels of Bip, p-eIF2α, LC3II/I, p62 and ALP were shown. **f** Partial silencing of eIF2α protein level by eIF2α siRNA, and protein expression of p-eIF2α, LC3II/I, p62, and ALP in osteoblasts differentiated from bone marrow cells. The experiment was conducted in triplicate. The asterisks (*, **, and ***) represent *P* *<* 0.05, *P* *<* 0.01, and *P* *<* 0.001, respectively (*n* = 9).

Electron microscopy was employed to evaluate morphology of the ER in osteoblasts in the distal metaphysis of the femurs. In OVX animals, we observed significant expansion of the rough ER and a decrease in the number of ribosomes on the ER membrane. Compared to the sham group (8.97 ± 1.09%), OVX mice (19.65 ± 2.71%) increased the percentage of the rough ER area (*P* *<* 0.01), while salubrinal-treated OVX mice exhibited restoration of the expanded rough ER (11.40 ± 0.69%, *P* *<* 0.01; Fig. 3a).

Autophagy is characterized morphologically by the accumulation of acidic vesicular (AVs), and we examined AVs of osteoblasts in the distal metaphysis of the femurs using transmission electron microscopy. The number of autophagosomes in the OVX group was fewer than that in the sham group, but the number of osteoblast AVs was significantly increased in the salubrinal-treated group (*P* *<* 0.001; Fig. 3b).

### Promotion of osteogenesis by regulating ER stress and autophagy

IHC staining of femur sections showed that the levels of ATF4 was decreased in osteoblasts of OVX mice compared to those of the sham mice, while the level of CHOP was increased. In the salubrinal-treated group, the expression of ATF4 was increased and the level of CHOP was decreased (Fig. 3c, d).

Western blot analysis showed that salubrinal increased the levels of Bip, p-eIF2α, LC3II/I, and ALP, while it decreased p62 in the femur samples (Fig. 3e and Supplementary Fig. S1). To investigate the role of eIF2α in salubrinal’s action, the markers for the ER stress and autophagy were examined in osteoblasts differentiated from bone marrow cells with and without transfection of eIF2α siRNA. The result showed that transfection of eIF2α siRNA suppressed salubrinal’s effect on p-eIF2α, LC3II/I, p62, and ALP in bone marrow cells (Fig. 3f and Supplementary Fig. S2).

### Induction of LC3 through regulating Atg7

**Fig. 4:**
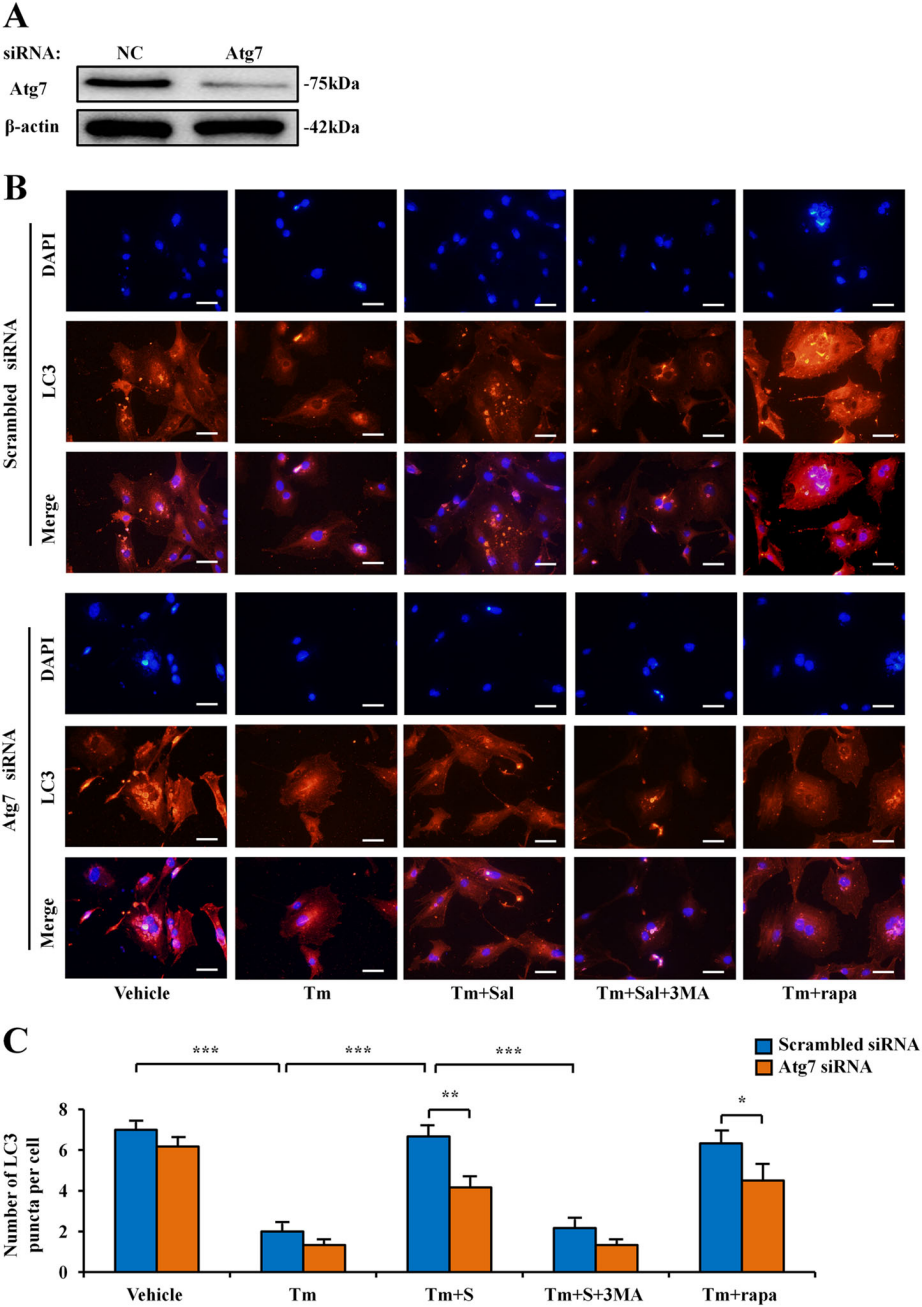
Induction of the LC3 puncta through regulating Atg7 by salubrinal. **a** Partial silencing of Atg7 protein level by Atg7 siRNA. **b** Representative immunofluorescence images of LC3 in MC3T3-E1 cells from different groups. (blue: DAPI; red: LC3^+^ cells; Tm: tunicamycin; Tm + Sal: tunicamycin + salubrinal; Tm + 3-MA: tunicamycin + 3-MA; Tm + rapa: tunicamycin + rapamycin; 400×, Bar = 50 μm). **c** Quantification of LC3 puncta per cell is shown. The experiment was conducted in triplicate. The asterisks (*, **, and ***) represent *P* *<* 0.05, *P* *<* 0.01, and *P* *<* 0.001, respectively.

To further evaluate the mechanism by which salubrinal regulates autophagy in the differentiated osteoblasts from MC3T3-E1 cells (Supplementary Fig. S3a, b), we evaluated the effect of siRNA specific to Atg7 (Fig. 4a). We also employed tunicamycin (ER stress inducer), salubrinal, 3-MA and rapamycin (inhibitor and stimulator of autophagy, respectively), and detected the level of an autophagy marker protein, LC3.

Compared to the vehicle group, the number of LC3 puncta was decreased after treatment with tunicamycin, while the autophagic puncta was significantly increased after salubrinal treatment. As expected, 3MA decreased LC3, and rapamycin increased LC3. When MC3T3-E1 cells were treated with siRNA specific to Atg7, salubrinal-driven alteration of LC3 was diminished compared to non-specific controls. Taken together, the observed expression pattern of LC3 was consistent with the role of salubrinal (Fig. 4b, c). Compared to the vehicle, salubrinal and rapamycin (*P* < 0.05) promoted the LC3 puncta, while 3MA significantly inhibited the autophagic fluorescent signal (*P* < 0.001; Supplementary Fig. S4a, b).

**Fig. 5:**
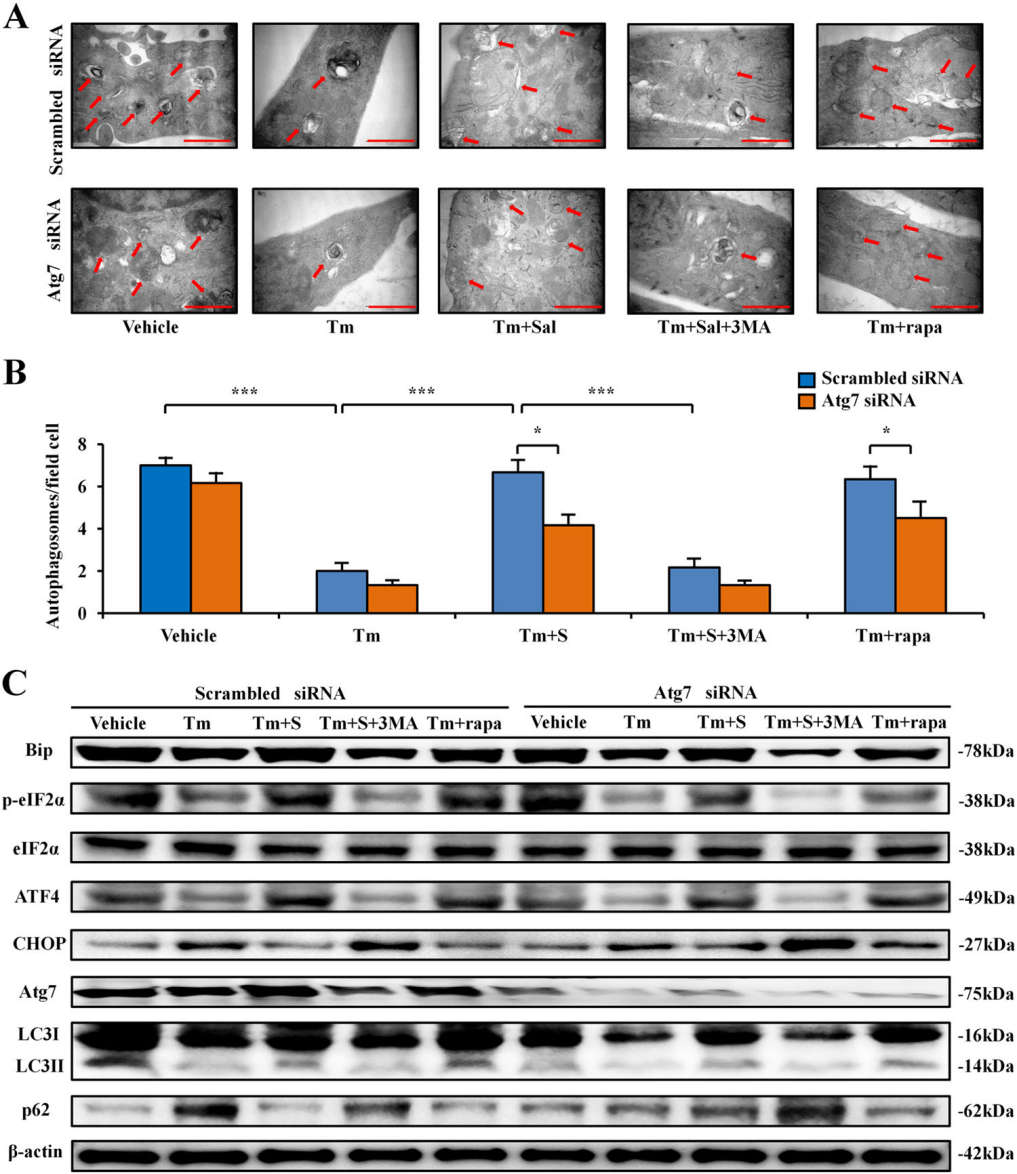
Regulation of ER stress and autophagy through Atg7 by salubrinal. **a, b** Representative electron micrographs of accumulation of autophagic vacuoles in the differentiated osteoblasts from MC3T3-E1 cells. Red arrow indicated autophagosomes. **c** Representative images of Western blot in different groups in vitro. The levels of Bip, eIF2α, ATF4, CHOP, Atg7, LC3II/I, and p62 were shown. The experiment was conducted in triplicate. The asterisks (*, **, and ***) represent *P* *<* 0.05, *P* *<* 0.01, and *P* *<* 0.001, respectively.

Compared to the vehicle group, the number of AVs was decreased after treatment with tunicamycin, while the autophagosomes were significantly increased after salubrinal treatment. However, 3MA decreased the number of autophagosomes, and rapamycin increased them. When cells were treated with siRNA specific to Atg7, salubrinal-driven alteration of autophagosomes was significantly diminished compared to the cells treated with non-specific control siRNA. Taken together, the observed expression pattern of autophagosomes was consistent with the role of salubrinal (Fig. 5a, b).

Compared to the vehicle group, western blot analysis showed that tunicamycin induced endoplasmic reticulum stress, which significantly changes expression of Bip, eIF2α, ATF4, and CHOP, while salubrinal restored expression of Bip, eIF2α, ATF4, and CHOP. The result showed that transfection of Atg7 siRNA slightly blocked the effect of salubrinal on expression of Bip, eIF2α, ATF4, and CHOP. While partially silencing Atg7, the expression of LC3 and p62 was not significantly attenuated by regulating the expression of the autophagy-associated protein Atg7. These results indicated that salubrinal promoted eIF2α-driven osteoblast autophagy through regulating the expression of Atg7 (Fig. 5c and Supplementary Fig. S5).

### Bone resorption and osteoclastogenesis

**Fig. 6:**
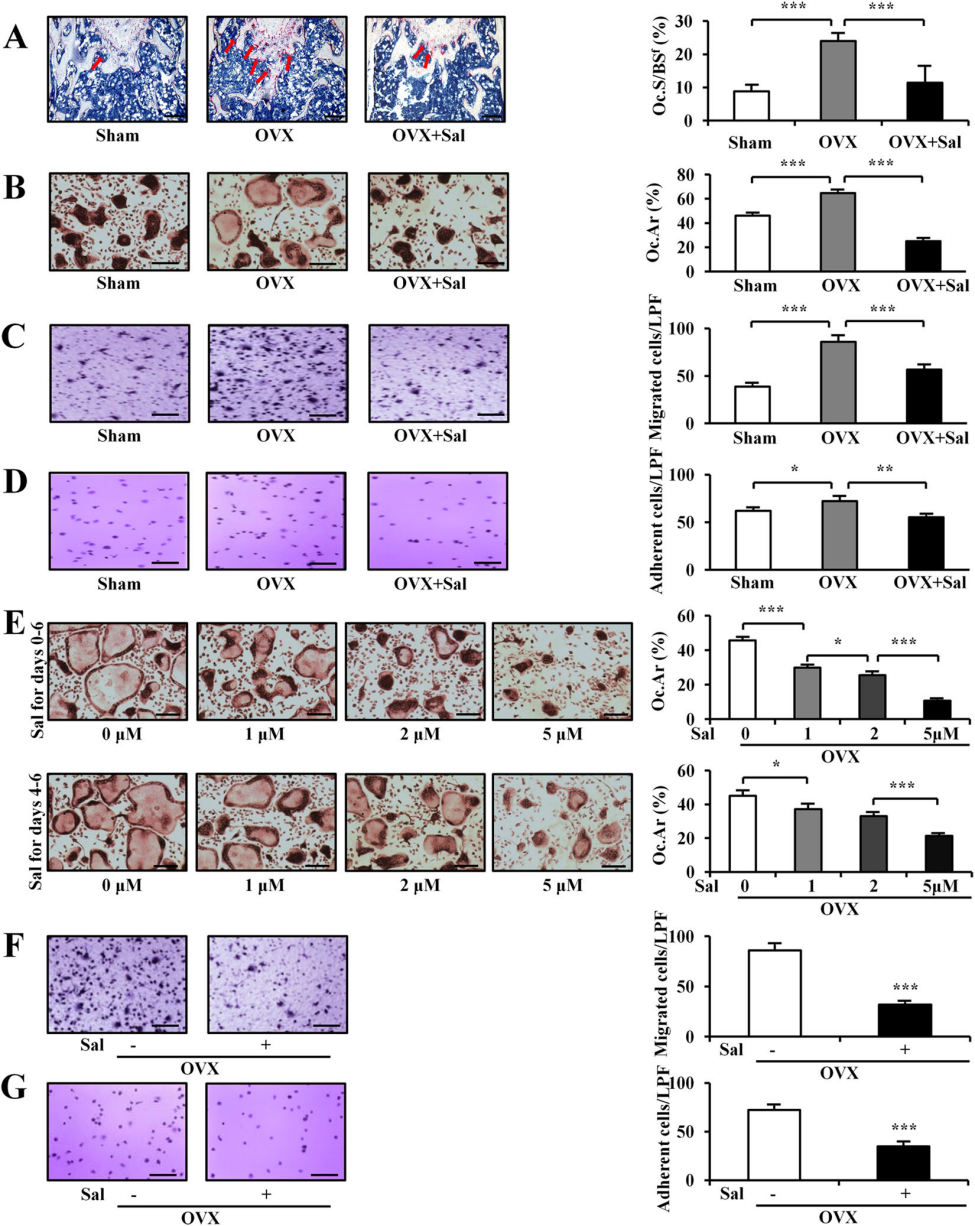
Effects of salubrinal on osteoclast development. **a** The osteoclast number in the OVX group was significantly suppressed by salubrinal injection. The representative photographs are shown (Bar = 200 μm). TRAP-positive cells in the distal metaphysis of the femurs, red color, indicated by the arrows. **b** Osteoclast formation was conducted using bone-marrow-derived cells. Coverage area of mature osteoclasts. **c, d** Osteoclast function was evaluated through migration and adhesion assays. Migration assay of osteoclasts (**c**). Adhesion assay of osteoclasts (**d**). **e** Coverage area of mature osteoclasts. Salubrinal was applied at 3 doses (1, 2, and 5 µM) and 2 phases (on days 0 and 4) to bone-marrow-derived cells isolated from OVX mice. Four pairs of images on the bottom show representative osteoclasts stained with TRAP. (Bar = 200 µm). Experiments were conducted in triplicates. Five fields were counted per well. Upper panel: Effects of in vitro salubrinal administration during days 0–6. Bottom panel: Effects of in vitro salubrinal administration during days 4–6. **f** Salubrinal-induced reduction in the migration of osteoclasts in both Sham-derived cells and OVX-derived cells. **g** Salubrinal-induced suppression of the adhesion of osteoclasts in both Sham-derived cells and OVX-derived cells. The asterisks (*, **, and ***) represent statistical significance at *P* *<* 0.05, *P* *<* 0.01, and *P* *<* 0.001, respectively (*n* = 12).

Compared to the sham control, TRAP staining of the distal metaphysis of the femurs showed that the osteoclast surface (Oc.S/BS^f^) was significantly increased in OVX mice (*P* *<* 0.001). However, the elevated ratio was markedly reduced by salubrinal (*P* *<* 0.001; Fig. 6a).

Osteoclast formation was conducted using bone-marrow-derived cells. The surface area (index of mature multinucleated osteoclasts formation) of mature osteoclasts was increased in the OVX mice, whereas a significant reduction was observed in the osteoclast surface area of salubrinal-treated OVX mice (both *P* *<* 0.001; Fig. 6b).

Osteoclast function was evaluated through migration and adhesion assays using bone-marrow-derived cells. The OVX group elevated both migration (*P* *<* 0.001; Fig. 6c) and adhesion (*P* *<* 0.05; Fig. 6d), while salubrinal-treated OVX mice significantly attenuated both migration (*P* *<* 0.001; Fig. 6c) and adhesion (*P* *<* 0.01; Fig. 6d).

### Dose-dependent effect on osteoclast differentiation

To further examine salubrinal’s effects on the maturation of osteoclasts, salubrinal was applied at three doses (1, 2, and 5 µM) and 2 phases (on days 0 and 4) to bone-marrow-derived cells isolated from OVX mice. Compared to the vehicle control, salubrinal for 6 days (days 0–6) significantly decreased the surface area covered by multinucleated osteoclasts (*P* *<* 0.001 for 0 µM vs 1 µM, *P* *<* 0.05 for 1 µM vs 2 µM; and *P* *<* 0.001 for 2 µM vs 5 µM). To test the effects of salubrinal on the late development, salubrinal was administered on days 4–6. This shorter incubation with salubrinal was also capable of inducing salubrinal-driven decrease in the osteoclastic surface area in a dosage-dependent manner (*P* *<* 0.05 for 0 µM vs 1 µM, and *P* *<* 0.001 for 2 µM vs 5 µM; Fig. 6e). This timeline was also able to provide a significant decrease in osteoclast formation in dosage-dependent manner in the early and late phases in salubrinal-treated OVX mice.

We examined salubrinal’s effect using pre-osteoclast cells isolated from OVX mice. Salubrinal provided a slower migration rate (*P* *<* 0.001; Fig. 6f), and it induced a significant reduction in cell adhesion (*P* *<* 0.001; Fig. 6g).

### Effects of salubrinal on NFATc1 and Rac1

**Fig. 7:**
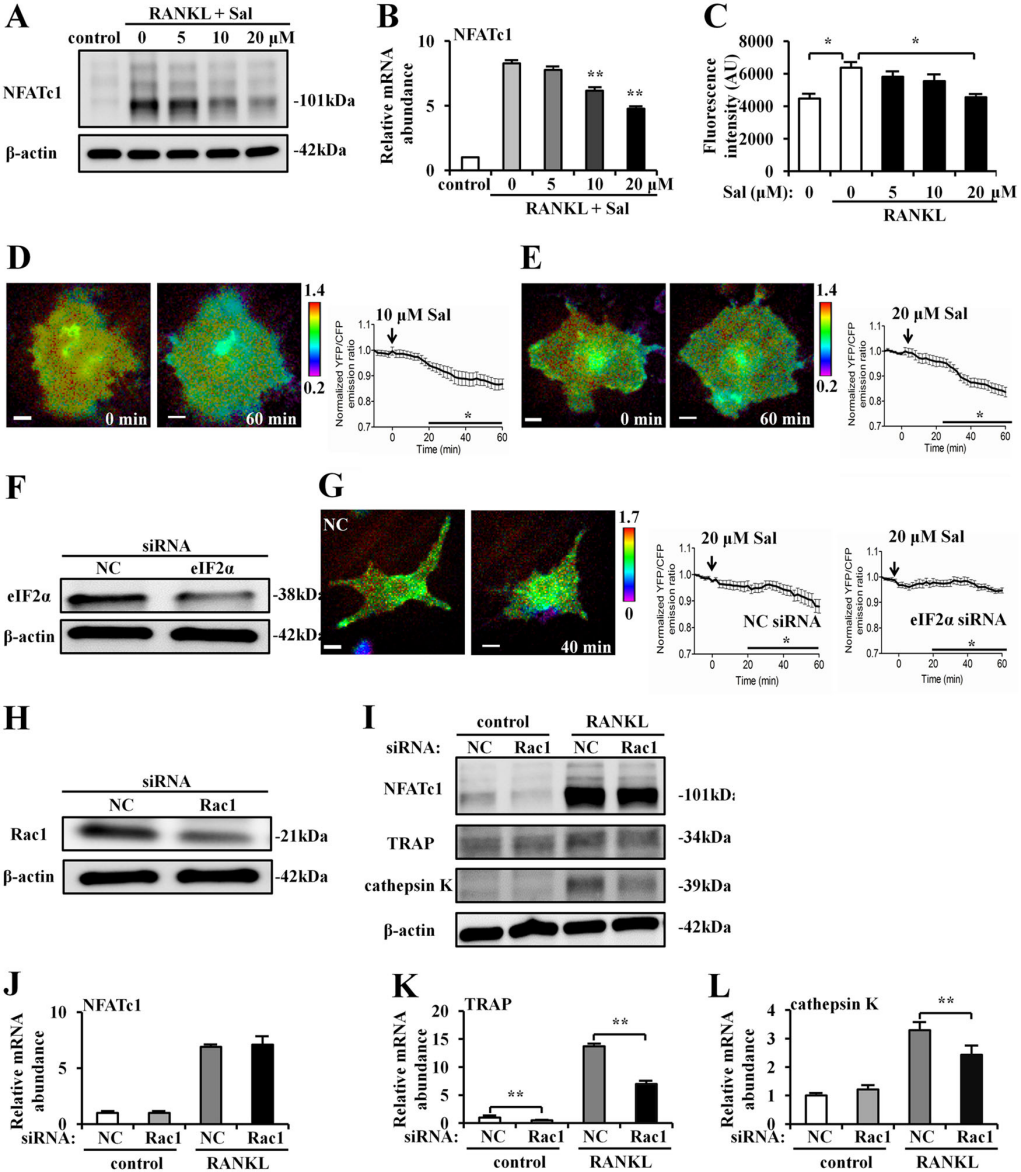
Effects of salubrinal on NFATc1 and Rac1. **a** Dose-dependent decrease in NFATc1 in RAW264.7 cells by 5–20 μM salubrinal. **b** Reduction of the mRNA levels of NFATc1 in RAW264.7 cells by 5–20 μM salubrinal. **c** Reduction in fluorescence-based osteoclast activity by salubrinal administration. **d, e** YFP/CFP emission ratios were averaged over the whole cell and normalized to time-10 min (10 min before Sal treatment). (bar = 10 μm). **f** Partial silencing of eIF2α protein level by eIF2α siRNA. **g** The activity of Rac1 in response to 20 μM salubrinal. NC = treatment with non-specific control siRNA. **h, i** Partial silencing of Rac1 protein level by Rac1 siRNA, and protein expression of NFATc1, TRAP, and cathepsin K. NC = treatment with non-specific control siRNA. **j, k** Relative mRNA levels of NFATc1, TRAP, and cathepsin K in response to Rac1 siRNA. TRAP and cathepsin K expressions are reduced by Rac1 siRNA, but NFATc1 expression remains unchanged. The experiment was conducted in triplicate. The asterisks (*, **, and ***) represent statistical significance at *P* *<* 0.05, *P* *<* 0.01, and *P* *<* 0.001, respectively.

To gain insight into the mechanism underlying the action of salubrinal on osteoclast development, migration, and adhesion, the effect of salubrinal on RAW264.7 monocyte/macrophage cells was evaluated. Western blot analysis showed that salubrinal decreased the protein level of the transcription factor critical for osteoclast differentiation, NFATc1, in a dose-dependent fashion (Fig. 7a). Administration of salubrinal (5, 10, and 20 μM) reduced RANKL-driven elevation of NFATc1 mRNA level in a dose-dependent manner (Fig. 7b). Furthermore, a fluorescence-based osteoclast activity assay showed that RANKL-driven increase in osteoclast activity was decreased by administration of salubrinal (*P* *<* 0.05 for 20 µM vs 0 µM; Fig. 7c).

To examine the effect of salubrinal on Rac1 GTPase activity in RAW264.7 cells, we used FRET-based live cell imaging. The emission ratio of YFP/CFP was reduced after administration of 10 and 20 μM salubrinal, supporting salubrinal’s inhibitory role in Rac1 activation (Fig. 7d, e). When RAW264.7 cells were transfected with siRNA specific to eIF2α, salubrinal-driven downregulation of Rac1 activity was significantly diminished (*P* *<* 0.05; Fig. 7f, g).

Using siRNA specific to Rac1 in RAW264.7 cells, the role of Rac1 in expression of NFATc1, TRAP, and cathepsin K was examined. The result showed that TRAP and cathepsin K were downregulated by salubrinal. Consistent with salubrinal’s effects on those protein levels, a partial silencing of Rac1 suppressed RANKL-driven upregulation of TRAP mRNA and cathepsin K mRNA but did not alter the level of NFATc1 mRNA (Fig. 7h–l).

## Discussion

Our data showed that salubrinal prevented bone loss by promoting osteoblast differentiation and inhibiting osteoclast development in OVX mice. Salubrinal administration markedly suppressed OVX-induced bone loss in the lumbar spine, femur, and tibia. Consistent with its effects on BMD and BMC, salubrinal improved OVX symptoms, including a decrease in BV/TV, trabecular number, trabecular thickness and serum PINP, as well as an increase in trabecular spacing, body weight and serum TRAP-5b. Similarly, salubrinal effectively prevented OVX-driven inhibition of mineral apposition rates, MS/BS and BFR/BS.

This study demonstrates that the ER stress-autophagy axis plays a critical role in the mouse model of postmenopausal osteoporosis. Electron microscopy showed that OVX-induced enlargement of the rough ER and a decrease in the ribosome population on the osteoblastic ER membrane. Salubrinal suppressed OVX-induced expansion of the rough ER. IHC staining showed that salubrinal attenuated expression of ATF4 and CHOP in OVX mice. In bone marrow cells isolated from OVX mice, we also observed a decrease in the ratio of LC3II/I, and accumulation of p62. Immunofluorescence, electron microscopy analysis, and western blot analysis of LC3 indicated that salubrinal promoted autophagy of osteoblasts by regulating the expression of Atg7. LC3II is a marker of autophagic vacuoles and both LC3I and LC3II are biomarkers in monitoring autophagy^49^, and p62 serves as an indicator of autophagic degradation^50^. Collectively, the in vitro and in vivo results showed that salubrinal restores bone homeostasis in postmenopausal osteoporosis via simultaneous regulation of the ER stress and autophagy in bone-marrow-derived cells and osteoblasts.

When the ER stress becomes excessive, cells may initiate autophagy that potentially induces large-scale cellular degradation^51^. Autophagy may serve as a pro-survival mechanism in response to excessive cellular stress^52^. Autophagy is characterized morphologically by the accumulation of acidic vesicular, and we observed that the number of AVs in the OVX group were fewer than that in the sham group. The result showed that osteoblastic AVs were significantly increased in the salubrinal-treated group.

The results of CFU-F and CFU-OBL were consistent with salubrinal-driven promotion of bone formation in OVX mice. The increase in CFU-F by salubrinal suggested MSCs proliferation is stimulated in bone-marrow-derived cells, while the increase in ALP-positive cells in CFU-OBL indicated enhancement of osteoblast development. Alizarin red staining showed that the salubrinal-treated OVX mice promoted mineralization of osteoblasts. Salubrinal preserves eIF2α phosphorylation as a selective inhibitor of a phosphatase complex of eIF2α, protein phosphatase 1 (PP1)^17,53^. It is reported that the elevated level of p-eIF2α can accelerate the healing of bone wounds and enhance bone formation^54–56^. Salubrinal prevented apoptosis of osteoblasts induced by glucocorticoids and the concomitant decrease in bone mass and bone formation^57^. Our findings agree with this report, showing that salubrinal improves OVX-induced bone loss by regulating p-eIF2α, ATF4, CHOP, and LC3. It also contributes to suppress cellular stress, such as the stress to the ER, radiation, and unloading, by reducing translational efficiency in general.

Studies indicate that autophagy also plays an important role in bone resorption by osteoclasts. It has recently been observed in mice that pharmacological and genetic inhibition of autophagy reduces osteoclastogenesis and bone resorption, suppressing bone loss caused by ovariectomy or glucocorticoid treatment^58^. However, whether salubrinal regulates the development of osteoclasts through the regulation of autoregulation-associated protein Atg7 requires further study. Regarding the development of osteoclasts in the OVX mice, subcutaneous injection of salubrinal in vivo as well as incubation with salubrinal in vitro suppressed the proliferation of pre-osteoclasts as well as their differentiation to mature multinucleated osteoclasts^43^. Western blot analysis revealed that salubrinal reduced the expression of NFATc1 in RANKL-stimulated bone-marrow-derived cells as well as RAW264.7 cells. Because of p-eIF2α in reducing translational efficiency, regulation of NFATc1 by salubrinal is likely to be achieved at least in part at the level of translation^56^. Consistent with the previous study^43^, we observed, that both the mRNA and protein levels of NFATc1 were reduced by salubrinal. These results, together with previous studies^59^, suggest a mechanism for osteoclast regulation by salubrinal through p-eIF2α and NFATc1. Further studies will establish other genes that may mediate the response of NFATc1 to salubrinal treatment.

Live cell imaging using RAW264.7 cells revealed that salubrinal suppressed activity of Rac1 GTPase, which is involved in various functions including cell migration. Previous studies showed that salubrinal reduces proliferation and migration of breast cancer cells in a murine model of mammary tumor by eIF2α-mediated suppression of Rac1 GTPase^18^. Administration of salubrinal presented significant reduction in cell migration and adhesion in RANKL-injected mice^43^. Our previous study indicated that salubrinal inhibited migration and adhesion of osteoclast in a disuse osteoporosis animal model^10^. The previous study also demonstrated that salubrinal prevented the glycosylation of Rac1 caused by eIF2α signaling pathway^60^. Based on the result with eIF2α siRNA, this downregulation was in part mediated by eIF2α signaling. The observed result is consistent with salubrinal’s action on inhibiting migration of pre-osteoclast cells. Furthermore, RNA silencing with siRNA specific to Rac1 showed that Rac1 also mediates salubrinal-driven downregulation of TRAP and cathepsin K although expression of NFATc1 is not directly regulated by Rac1. This results demonstrate the potential of salubrinal as a therapy for reversing bone loss from osteoporosis. The mechanism of salubrinal was explored on bone cells using FRET and RNA inhibition to identify Rac1 GTPase as a mediator of its osteoclast suppression separate from NFATc1.

**Fig. 8:**
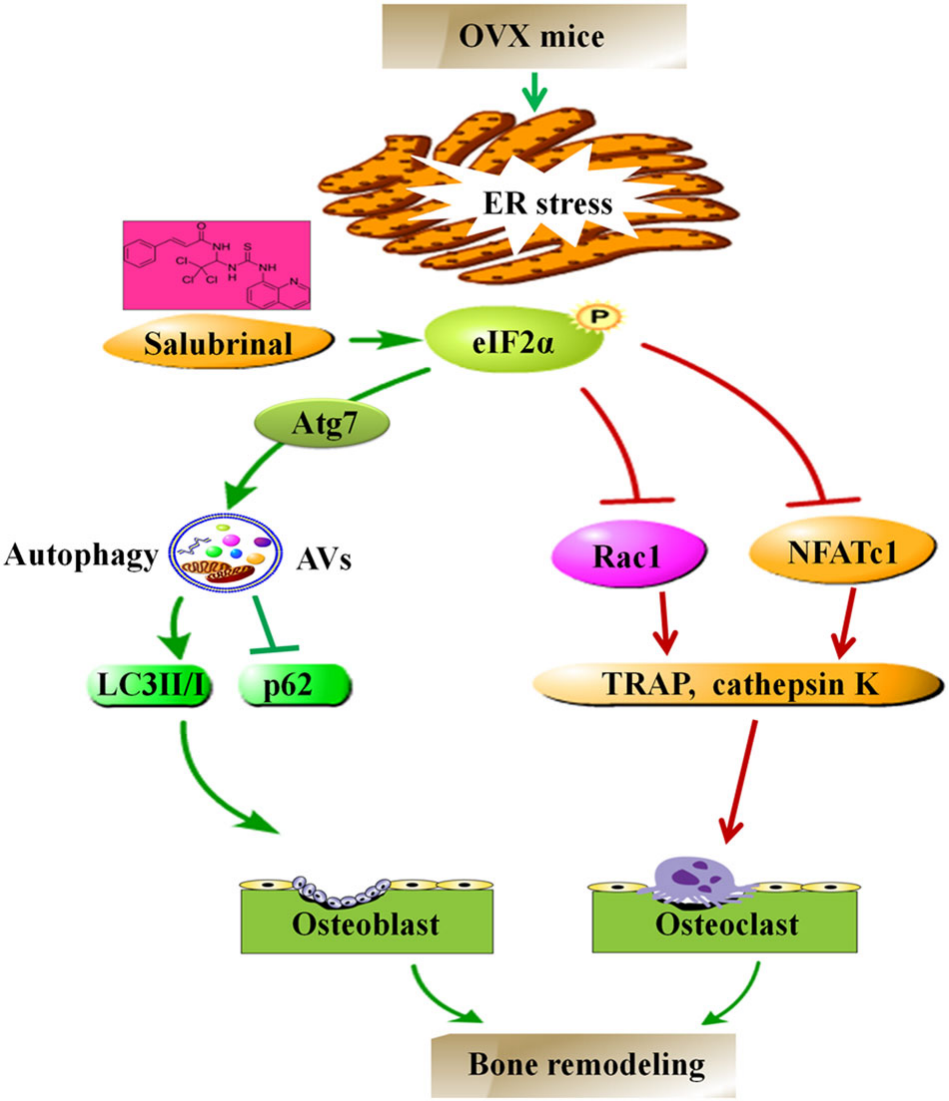
Proposed mechanism of salubrinal’s action in bone remodeling. Salubrinal attenuates the bone loss by regulating the ER stress-autophagy axis through stimulating the expression of Atg7 of osteoblasts and altering the proliferation and differentiation of osteoclasts by regulating eIF2α, Rac1 and NFATc1.

This study demonstrates that salubrinal attenuates the bone loss by regulating the ER stress-autophagy axis through stimulating the expression of Atg7 of osteoblasts and altering the proliferation and differentiation of osteoclasts by regulating eIF2α, Rac1, and NFATc1 (Fig. 8). This study presented the possibility of a drug therapy to prevent bone loss. The results herein utilizing the OVX mouse model and primary bone-marrow-derived cells, as well as cell line-based cultures, show that salubrinal prevented OVX-associated symptoms through stimulating osteoblasts development and inhibiting osteoclasts activity. Further analysis may focus on the underlying mechanism of salubrinal’s action warrants the possibility of a novel therapeutic strategy for postmenopausal osteoporosis.

## Supplementary information

Supplementary Information

Supplementary Figure S1

Supplementary Figure S2

Supplementary Figure S3

Supplementary Figure S4

Supplementary Figure S5

Supplementary Figure Legends
