# Supplementary material for: eIF2α signaling regulates autophagy of osteoblasts and the development of osteoclasts in OVX mice
Source: Cell Death Dis. 2019 Dec 4;10(12):921. doi: 10.1038/s41419-019-2159-z (PMC6892793; doi:10.1038/s41419-019-2159-z)
Supplement: Supplementary file 1 — Supplementary Information [file 41419_2019_2159_MOESM1_ESM.doc]

**Supplementary Information**

**eIF2α signaling regulates autophagy of osteoblasts and the development of osteoclasts in OVX mice**

**Jie Li, Xinle Li, Daquan Liu, Kazunori Hamamura, Qiaoqiao Wan, Sungsoo Na, Hiroki Yokota, and Ping Zhang**

**Supplementary Figure S**1

| **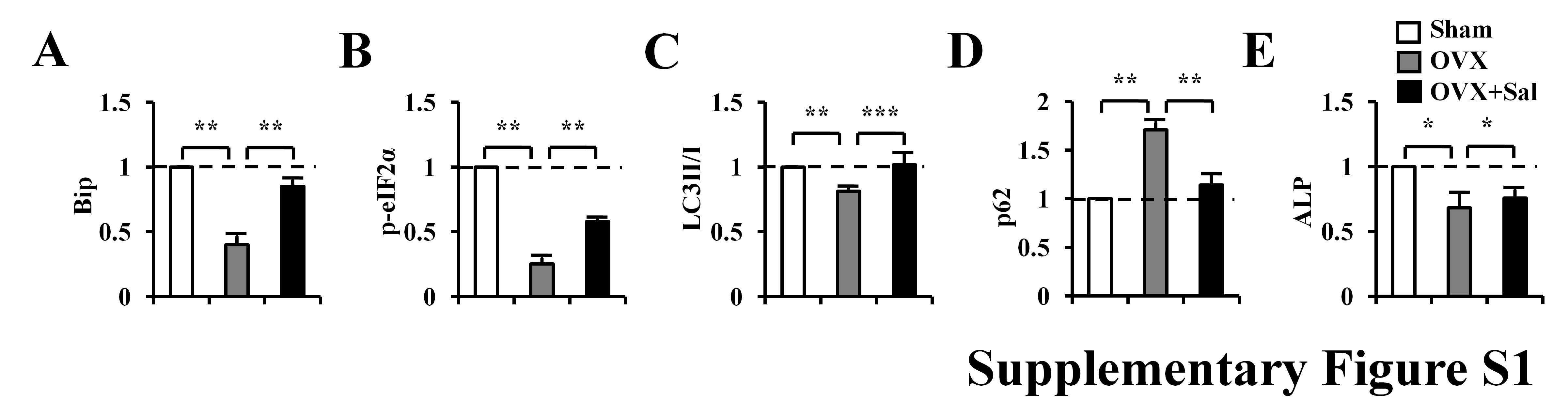** |
| --- |
| **Supplementary Figure S1:**  The data quantification of Western blot in three different groups *in vivo*. The levels of Bip, p-eIF2α, LC3II/I, p62 and ALP were shown. The experiment was conducted in triplicate. The asterisks (*, ** and ***) represent *P* < 0.05, *P* < 0.01 and *P* < 0.001, respectively (n = 3). |

**Supplementary Figure S2**

| **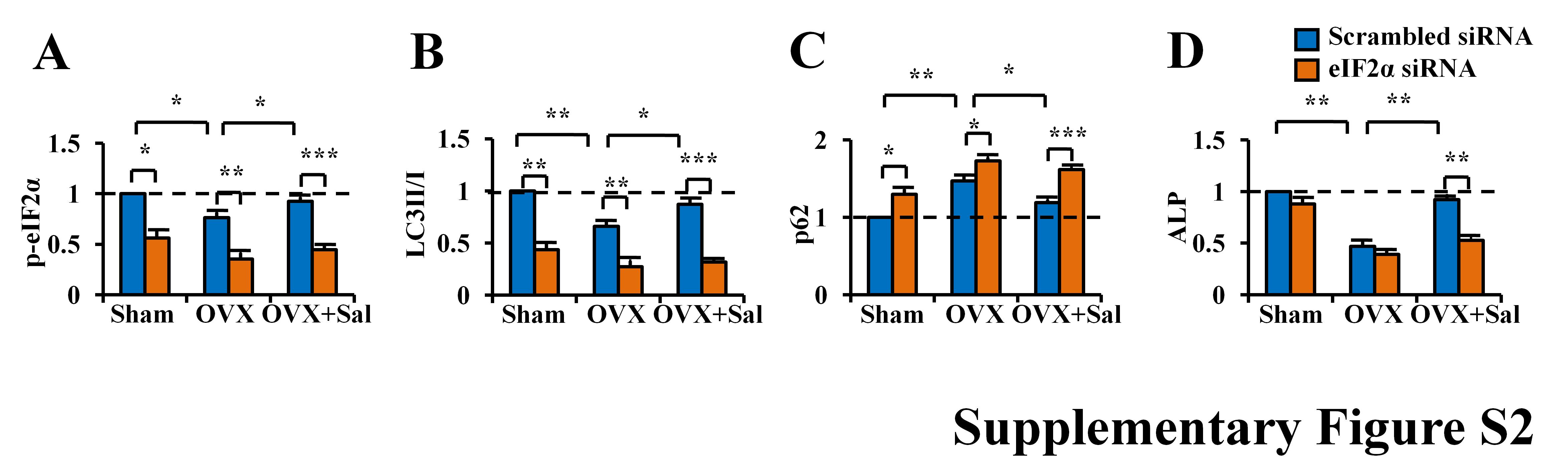** |
| --- |
| **Supplementary Figure S2:**  Partial silencing of eIF2α protein level by eIF2α siRNA, and protein expression of p-eIF2α, LC3II/I, p62 and ALP in osteoblasts differentiated from bone marrow cells. The experiment was conducted in triplicate. The asterisks (*, ** and ***) represent *P* < 0.05, *P* < 0.01 and *P* < 0.001. |

**Supplementary Figure S**3

| **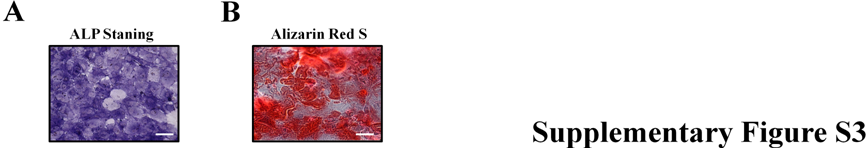** |
| --- |
| **Supplementary Figure S3:**  Representative images of ALP Staning (**a**) and Alizarin Red S (**b**) in differentiated osteoblasts from MC3T3-E1 cells. |

**Supplementary Figure S**4

| **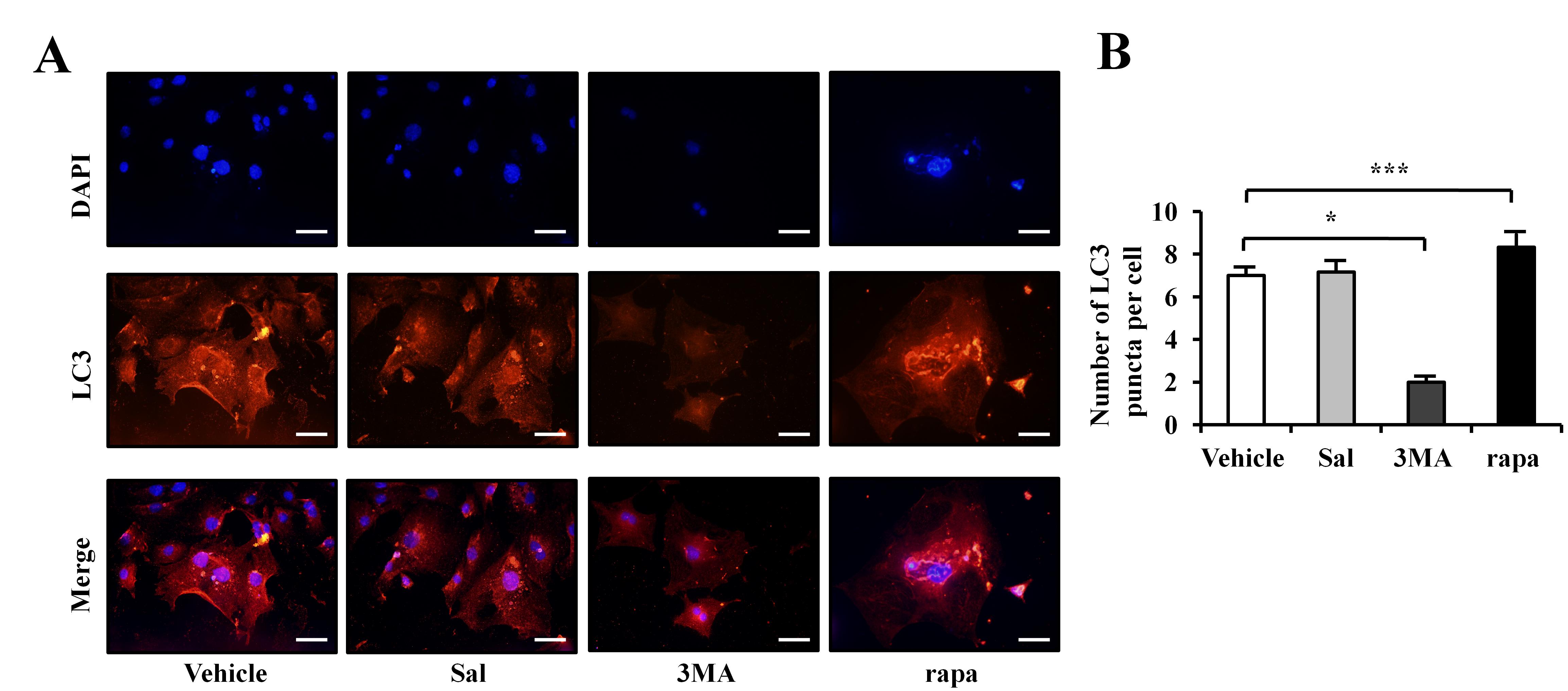** |
| --- |
| **Supplementary Figure S4:**  **a** Representative immunoﬂuorescence images of LC3 in differentiated osteoblasts from MC3T3-E1 cells from different groups. (blue: DAPI;, red: LC3^+^ cells; Sal: salubrinal; 3-MA; rapa: rapamycin; 400×, Bar = 50 μm). **b** Quantification of LC3 puncta per cell is shown. The experiment was conducted in triplicate. The asterisks (*, ** and ***) represent *P* < 0.05, *P* < 0.01 and *P* < 0.001. |

**Supplementary Figure S5**

| **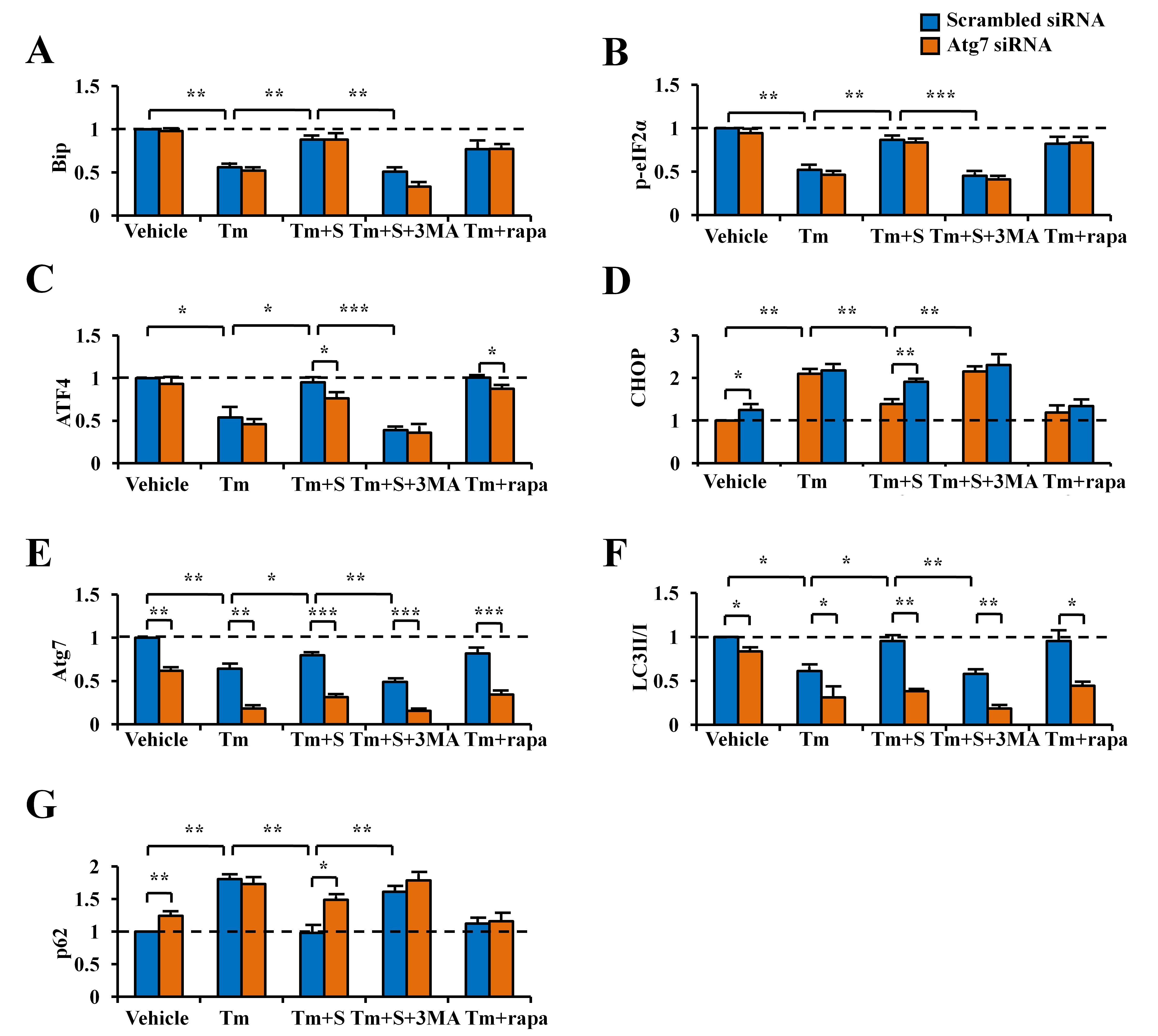** |
| --- |
| **Supplementary Figure S5:**  The data quantification of Western blot in differentiated osteoblasts from MC3T3-E1 cells from different groups. The levels of Bip, eIF2α, ATF4, CHOP, Atg7, LC3II/I and p62 were shown. The asterisks (*, ** and ***) represent *P* < 0.05, *P* < 0.01 and *P* < 0.001, respectively. |
