# Supplementary figures and images for: eIF2α signaling regulates autophagy of osteoblasts and the development of osteoclasts in OVX mice
Source: Cell Death Dis. 2019 Dec 4;10(12):921. doi: 10.1038/s41419-019-2159-z (PMC6892793; doi:10.1038/s41419-019-2159-z)

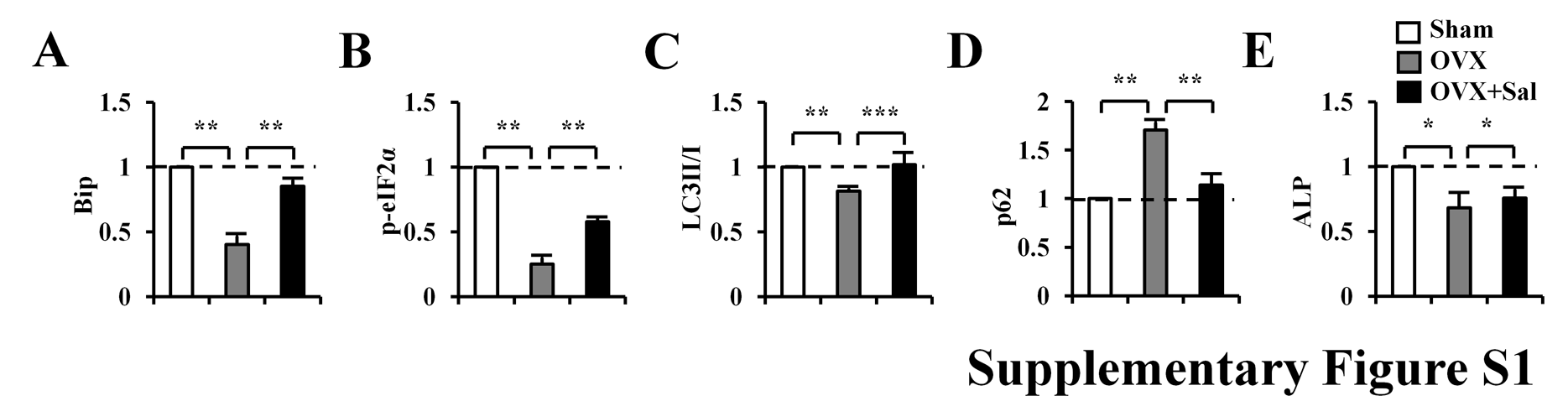

Supplement: Supplementary file 2 — Supplementary Figure S1 [file 41419_2019_2159_MOESM2_ESM.tif]

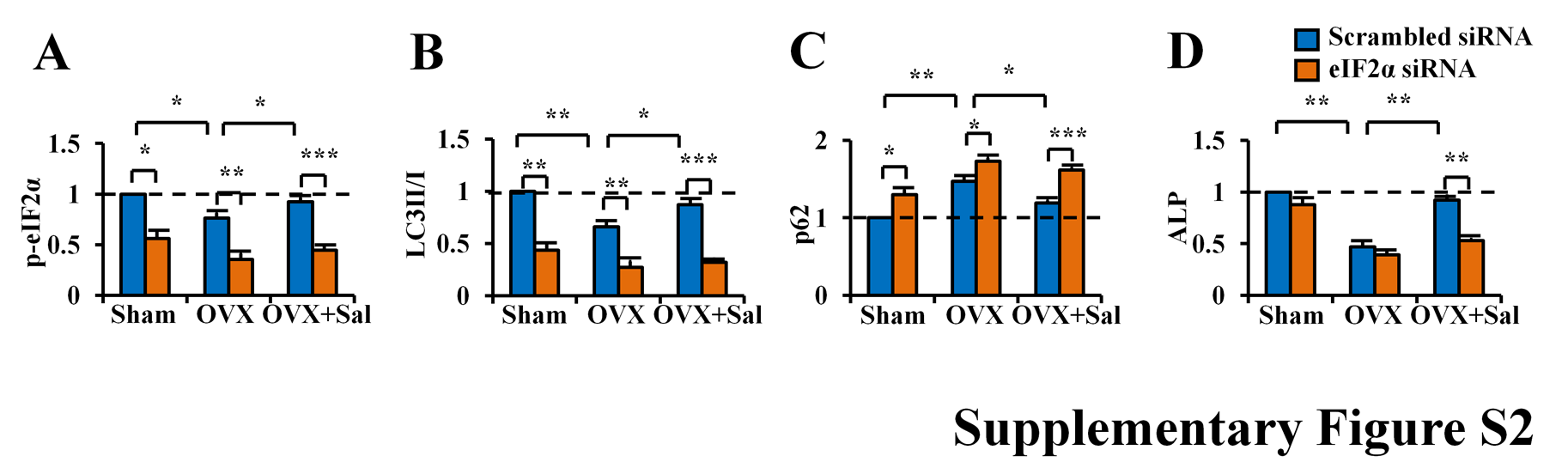

Supplement: Supplementary file 3 — Supplementary Figure S2 [file 41419_2019_2159_MOESM3_ESM.tif]

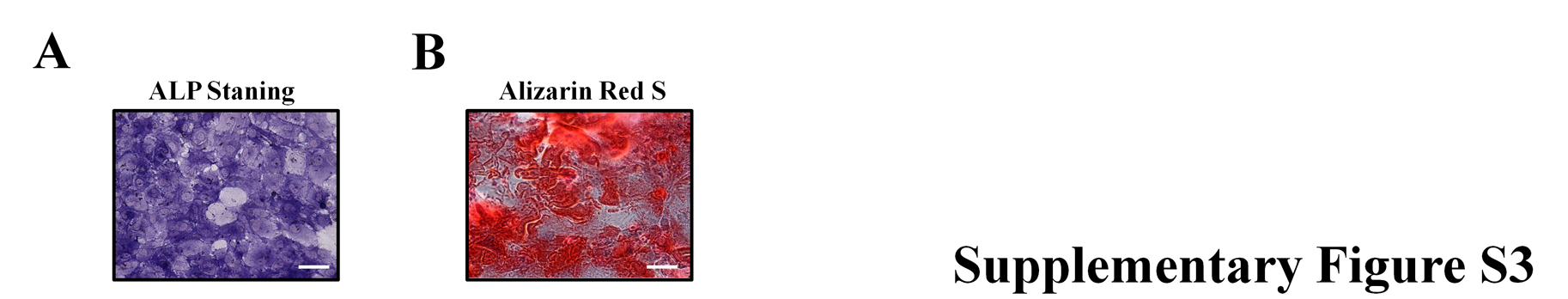

Supplement: Supplementary file 4 — Supplementary Figure S3 [file 41419_2019_2159_MOESM4_ESM.tif]

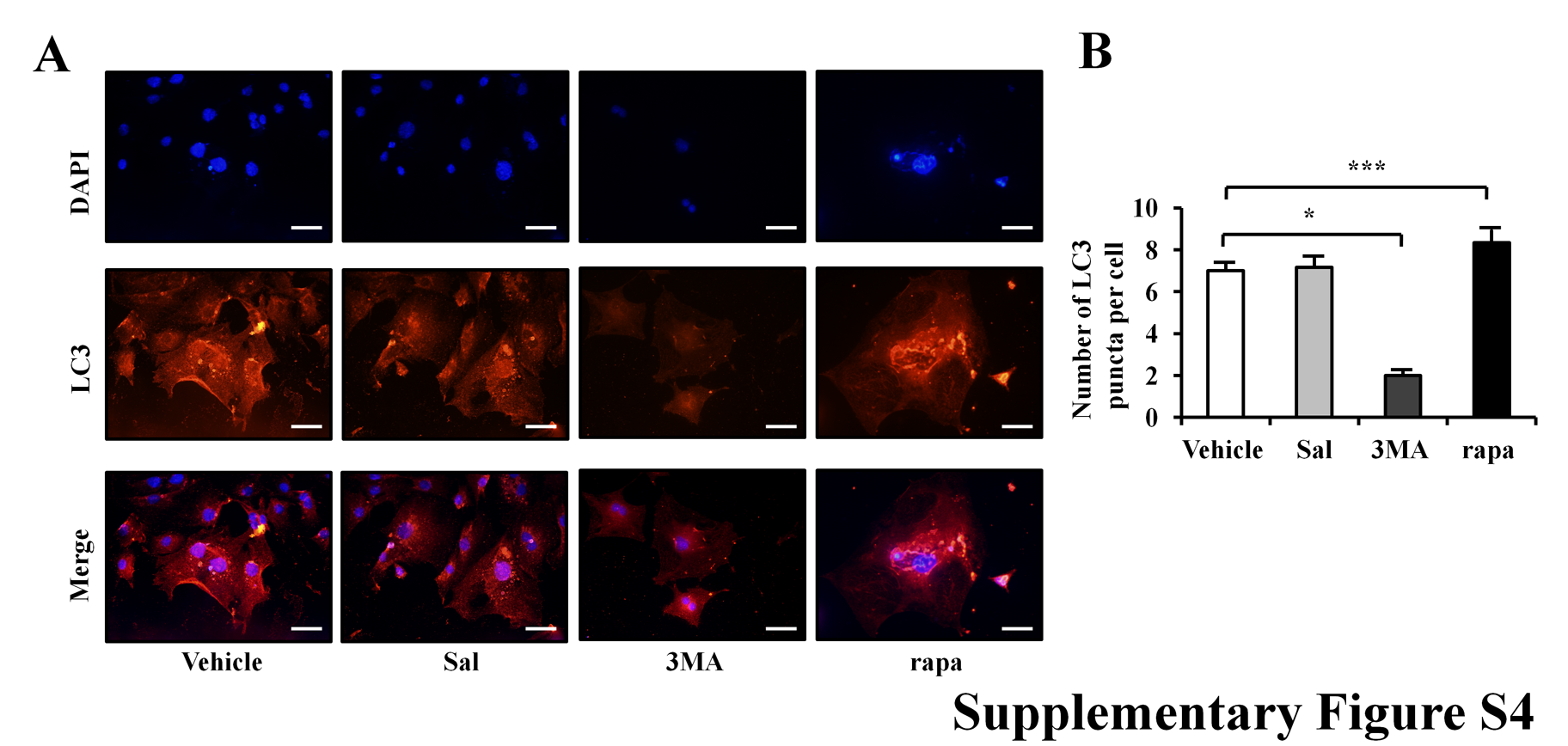

Supplement: Supplementary file 5 — Supplementary Figure S4 [file 41419_2019_2159_MOESM5_ESM.tif]

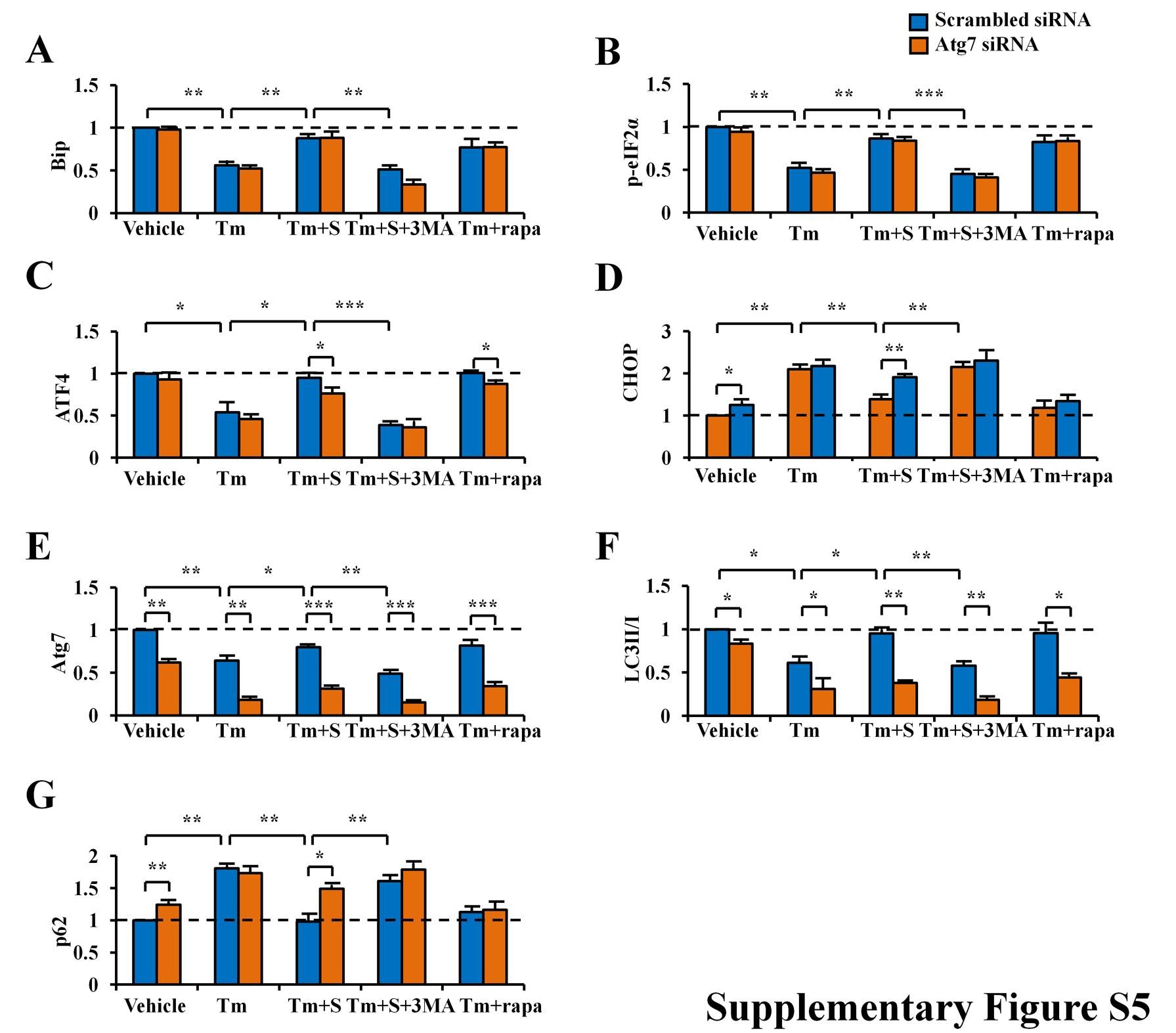

Supplement: Supplementary file 6 — Supplementary Figure S5 [file 41419_2019_2159_MOESM6_ESM.tif]
